# Brain representations of space and time in episodic memory: A systematic review and meta-analysis

**DOI:** 10.3758/s13415-023-01140-1

**Published:** 2023-11-29

**Authors:** César Torres-Morales, Selene Cansino

**Affiliations:** Laboratory of NeuroCognition, Faculty of Psychology, National Autonomous University of Mexico, Mexico City, Mexico

**Keywords:** Spatial context, Temporal context, Source memory, Recollection, fMRI, Meta-analysis

## Abstract

All experiences preserved within episodic memory contain information on the space and time of events. The hippocampus is the main brain region involved in processing spatial and temporal information for incorporation within episodic memory representations. However, the other brain regions involved in the encoding and retrieval of spatial and temporal information within episodic memory are unclear, because a systematic review of related studies is lacking and the findings are scattered. The present study was designed to integrate the results of functional magnetic resonance imaging and positron emission tomography studies by means of a systematic review and meta-analysis to provide converging evidence. In particular, we focused on identifying the brain regions involved in the retrieval of spatial and temporal information. We identified a spatial retrieval network consisting of the inferior temporal gyrus, parahippocampal gyrus, superior parietal lobule, angular gyrus, and precuneus. Temporal context retrieval was supported by the dorsolateral prefrontal cortex. Thus, the retrieval of spatial and temporal information is supported by different brain regions, highlighting their different natures within episodic memory.

## Introduction

Space and time have been the subject of intense debates in different disciplines. In the field of philosophy, Issac Newton conceived of space and time as absolute entities unrelated to anything external; thus, space was identical and immobile, and time flowed uniformly. Relative space and time refer to the ways that these variables are measured (Rynasiewicz, 1995). Gottfried Leibniz viewed space and time as completely relative rather than real entities. Space refers to the order of objects, and time refers to the order of successions (Evangelidis, 2018). In physics, the three Cartesian dimensions that define space cannot be disentangled from time, the fourth dimension, because object movement through space requires time, which depends on the object’s speed relative to the observer (Kennedy, 2003). Thus, the measurements of space and time, i.e., distance and duration, are linked to speed, and any of these variables can be derived from the other two. In the field of neuroscience, such debates are important, because it remains unknown whether we actually perceive and experience space and time or whether these experiences are created in our brain (Buzsáki & Llinás, 2017).

Moreover, in neuroscience, another debate is whether space and time are intrinsically related (i.e., the brain employs the same mechanisms and structures to process both experiences) or independent experiences (i.e., processed by different mechanisms and structures within the brain). Research has supported both points of view. The finding that specific neurons within the hippocampus are capable of processing different types of information supports the argument that space and time are independent experiences. For example, certain neurons located in the CA1 region of the anterior dorsal hippocampus respond only when a rat is situated in a specific location and direction; these neurons are called place cells (O’Keefe & Dostrrovsky, 1971). Space is further represented by grid cells located in the dorsocaudal medial entorhinal cortex that respond when a rat is located in the vertex of imaginary equilateral triangles spread across the floor surface (Hafting, et al., 2005). Several studies have provided evidence of the existence of time cells. One study (Pastalkova et al., 2008) reported that specific neurons in the CA1 respond in sequence while a rat is on a running wheel during the delay period of a T-maze task; neuronal activity differed according to whether the previous tasks involved a right or left turn. In humans, place cells (Ekstrom et al., 2003) and time cells (Umbach et al., 2020) also have been identified in the hippocampus through intracranial recordings in epileptic patients.

Evidence indicating that space and time are coded by the same neural mechanisms also has been obtained in the hippocampus. By modifying the speed of a treadmill, it was possible to disentangle the neural activity related to the distance run by a rat and the time that the rat spent on the treadmill (Kraus et al., 2013). Speed was randomly manipulated across trials: in some sessions, rats remained on the treadmill for the same amount of time across trials, and in other sessions, the rats ran the same distance on the treadmill across trials. Neurons that respond to time fired if the treadmill moved faster, because rats traveled a longer distance in the same amount of time. In contrast, neurons that code distance fired at faster speeds, because less time was needed to travel the same distance. This procedure demonstrated that most hippocampal neurons respond to both distance and time. In humans, functional magnetic resonance imaging (fMRI) studies using region of interest (ROI) analysis have revealed that the hippocampus encodes spatiotemporal context for memories that are distant in space and time. To examine the space and time of autobiographic experiences, participants automatically took photographs stamped with their spatial coordinates and time for one month. Then, 120 photographs were presented during fMRI scans, and participants were asked to mentally retrieve the experience and rate its vividness. The results of this study (Nielson et al., 2015) indicated that the neural distance activity within the left hippocampus was correlated with temporal and spatial distance. Another study (Deuker et al., 2016) found that activity in the right medial to anterior hippocampus was related to both the spatial and temporal distance between objects seen in a virtual city tour.

Notably, the processing of spatial and temporal information has been extensively studied within the hippocampus and other regions in the medial temporal lobe. However, the contribution of other neocortical regions to the processing of spatial and temporal information has been overlooked, even though several studies have identified cortical regions involved in spatial or temporal representations, such as the parietal cortex and the prefrontal cortex (Eichenbaum, 2017b). Moreover, an fMRI study (Schedlbauer et al., 2014) that employed graph theory analyses identified a network of several regions, including the hippocampus, prefrontal cortex, parietal cortex, and precuneus, that showed greater connectivity when participants correctly retrieved spatial and temporal contexts associated with two items encountered in a virtual tour in a city.

Regarding episodic memory, which refers to our ability to retrieve our own experiences (Tulving, 1972), space and time are the only two contextual attributes that are inherent to any episodic event, because all events occur in a specific location and moment. Therefore, when evaluating episodic memory performance, the retrieval of these contexts provides reliable evidence of episodic memory functioning. In memories, space and time are mentally represented along with the event itself; thus, they are not real entities but are reminiscent of real experiences. However, it remains unclear which brain regions (other than structures in the medial temporal lobe) are involved in the retrieval of spatial and temporal information and how these brain regions (along with structures in the medial temporal lobe) contribute to the retrieval of this critical information, because studies on this topic have been scattered. Therefore, the purpose of the present systematic review and meta-analysis was to integrate those findings and thereby provide a comprehensive understanding of how the brain retrieves spatial and temporal information linked to episodic memory experiences. Although several studies (Eichenbaum et al., 2007; Ekstrom & Ranganath, 2018; Howard, 2017; Lipton & Eichenbaum, 2008; Sugar & Moser, 2019) have reviewed how the brain processes spatial and temporal information in nonprimate and primate species, these studies focused mainly on the hippocampus. To the best of our knowledge, this is the first meta-analysis to examine fMRI and positron emission tomography (PET) studies that assessed the retrieval of spatial and temporal contexts in episodic memory.

We aimed to identify the brain regions that exclusively contribute to the retrieval of spatial information and those that support only the retrieval of temporal information. To identify the brain regions involved in the retrieval of spatial and temporal information, we included fMRI and PET studies that independently examined only one of these contexts or that examined both spatial and temporal contexts in the same study. Moreover, we included only studies that conducted whole-brain analyses and excluded ROI analyses to ensure equal potential contributions of all potential brain regions to be estimated within the meta-analyses. Furthermore, to isolate the brain regions associated with spatial or temporal context retrieval, we contrasted brain activity against a control condition, recognition, the opposite context or a combination of these conditions. This procedure allowed us to identify which brain regions are involved in each context more than any other cognitive process.

Because we were interested in identifying the brain regions that contribute to the retrieval of spatial and temporal information in healthy adults with optimal conditions of episodic memory, we excluded studies conducted in older adults and in adults affected by any psychiatric disorder or neurological disease. However, if studies were performed on older and younger adults, the results from younger adults were included in the meta-analyses. We expected that the meta-analyses would identify hippocampal regions as responsible for exclusive processing of spatial or temporal information, consistent with findings from electrophysiology studies conducted in rats (O’Keefe & Dostrrovsky, 1971; Pastalkova et al., 2008) and humans (Ekstrom et al., 2003; Umbach et al., 2020). Additionally, we anticipated that a hippocampal region would support the processing of both types of information, as reported in studies with rats (Kraus et al., 2013) and humans (Deuker et al., 2016; Nielson et al., 2015). Likewise, we expected that the parietal cortex and the prefrontal cortex would contribute to the retrieval of spatial or temporal information, as observed in previous studies (Eichenbaum, 2017b).

## Methods

We conducted a systematic review and meta-analysis according to the Preferred Reporting Items for Systematic Reviews and Meta-Analyses (PRISMA) guidelines (Moher et al., 2009). We included all fMRI or PET articles that examined episodic memory retrieval of spatial or temporal information in healthy young adults. The following databases were searched to identify relevant articles: PubMed, SpringerLink, PsycInfo, and Scopus. All relevant studies, regardless of publication date, were included. The search was performed with the following keywords: episodic memory, context memory, source memory, recollection, spatiotemporal context, spatial context, temporal context, spatiotemporal, spatial, temporal order, functional magnetic resonance imaging, fMRI, positron emission tomography, and PET. We identified additional articles by examining the references of the selected studies. Only articles published in English were included.

The inclusion criteria for studies were as follows: (1) used fMRI or PET; (2) examined brain activity associated with the retrieval of spatial or temporal information within episodic memory; (3) compared the retrieval of spatial or temporal information with a control condition, recognition, other context, or incorrect retrieval; (4) reported whole-brain analyses; (5) provided Montreal Neurological Institute (MNI) or Talairach & Tournoux (1988) stereotaxic coordinates; and (6) enrolled healthy young adults. The exclusion criteria were as follows: (1) reported only region of interest (ROI) analyses or (2) recruited older adults or individuals affected by psychiatric disorders or neurological diseases without reporting results in healthy young adults. Eligibility was determined independently by two reviewers. The current study did not have a risk of selection bias, because we included all articles on the subject.

For each included study, we extracted the following information: sample size, mean age and age range of participants, episodic memory task, imaging technique, contrasts analyzed, and brain neuroimaging results (x/y/z coordinates).

### Activation Likelihood Estimation

Activation likelihood estimation (ALE) meta-analyses were conducted for the following contrasts: (1) correct retrieval of spatial information versus a control condition, recognition, other context or incorrect retrieval; and (2) correct retrieval of temporal information versus a control condition, recognition, other context or incorrect retrieval. Meta-analyses were performed with GingerALE version 3.0.2 (Eickhoff et al., 2009, 2012; Turkeltaub et al., 2012). ALE meta-analysis allows the identification of activation foci from different experiments that are significantly higher than a null distribution of random spatial activations across the studies (Eickhoff et al., 2009). Coordinates reported in Talairach space were converted into MNI space using the tal2icbm transform (Lancaster et al., 2007) implemented in GingerALE. First, maximal activation foci were modeled as peaks of three-dimensional Gaussian probability distributions. The full-width at half maximum (FWHM) of the Gaussian function was automatically estimated from the number of subjects included in each experiment. Then, the foci reported in each experiment were modeled as activation maps. The combination of all activation maps yielded the ALE scores at every voxel. The ALE null distribution was created by randomly distributing the same number of foci across the whole brain. One thousand permutation tests were conducted to distinguish true converging clusters across studies from random clusters and to determine the statistical significance of ALE maps. The resulting ALE maps were thresholded using a cluster-level familywise error (FWE) correction at *p* < 0.05 and a cluster-forming threshold of *p* < 0.001 (Eickhoff et al., 2012, 2016). The program Mango (http://ric.uthscsa.edu/mango) was used to display the results, and significant ALE clusters were overlaid on the Colin template (Colin27_T1_seg_MNI.nii) provided by GingerALE (Eickhoff et al., 2009, 2012).

## Results

**Fig. 1:**
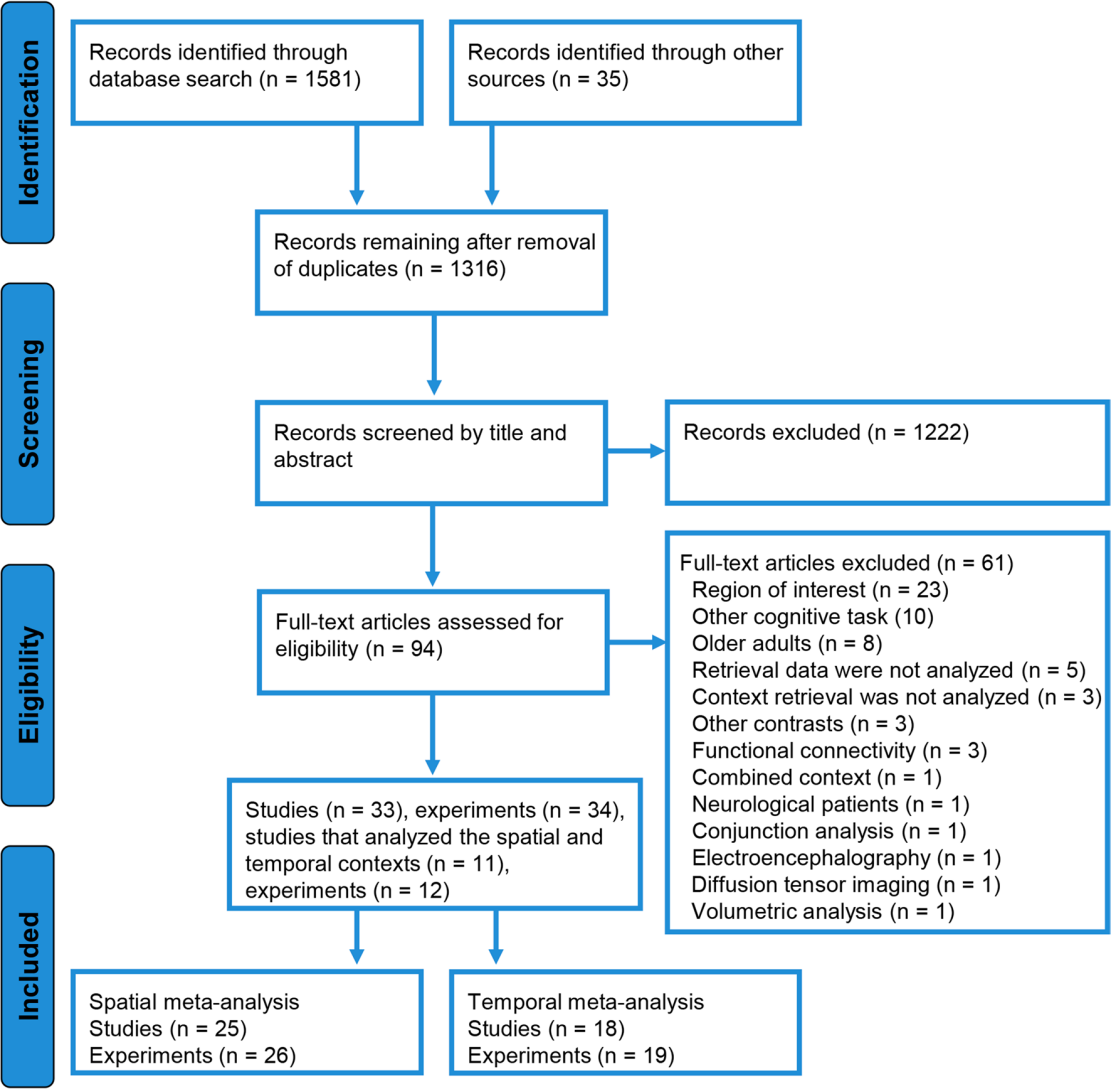
Study selection for the meta-analyses

A total of 26 experiments from 25 studies were eligible for inclusion in the spatial meta-analysis, and 19 experiments from 18 studies were included in the temporal meta-analysis. Figure 1 displays the flowchart of study selection for inclusion in both meta-analyses. The main characteristics of the included studies are displayed in Table 1, such as sample size and participant age (if the information was available). The numbers of participants included in the studies in the spatial and temporal meta-analyses were 369 and 264, respectively. Most studies employed fMRI, except for eight that used PET. The encoding phase took place outside the scanner in 15 of the experiments included in the spatial meta-analysis and in 14 of the experiments included in the temporal meta-analysis. Of the studies that conducted scans during the encoding phase, seven from the spatial meta-analysis analyzed these data, and three from the temporal meta-analysis analyzed these data. The memory tasks employed in each study are described in Tables 2, 3 and 4. Most studies included in the spatial meta-analysis employed images as stimuli, except for five that used words as stimuli. Conversely, ten of the studies included in the temporal meta-analysis used words as stimuli.

**Table 1:** Studies included in the spatial and temporal meta-analyses

	Meta-analysis	Sample size (n)	Age, yr Mean (range)	Imaging technique
Bergström et al., 2013	S	18	25.0 (19–35)	fMRI
Burgess et al., 2001	S	13	27.2	fMRI
Cabeza et al., 1997	T	12	25.0 (20–38)	PET
Cansino et al., 2002	S	17	24.6 (20–27)	fMRI
Cansino et al., 2015	S	12	23.2	fMRI
de Rover et al., 2008	S/T	20	25.0 (19–33)	fMRI
Dobbins et al., 2003	T	11	(19–26)	fMRI
Ekstrom & Bookheimer, 2007	S/T	14		fMRI
Ekstrom et al., 2011	S/T	16	21.5 (20–28)	fMRI
Frings et al., 2006	S	13	27.0 (21–39)	fMRI
Fujii et al., 2002	T	8	20.9 (19–25)	PET
Fujii et al., 2004	S/T	11	21.4 (19–24)	PET
Greve et al., 2010	T	17	23.0 (19–35)	fMRI
Hayes et al., 2004	S/T	14	24.0 (20–36)	fMRI
Hoscheidt et al., 2010	S	17	22.2 (18–30)	fMRI
Johnsrude et al., 1999	S	12	22.5 (18–33)	PET
King et al., 2005	S	13	26.9 (18–45)	fMRI
Kwok et al., 2012	S/T	15	25.9 (18–37)	fMRI
Kwok & Macaluso, 2015 E1	S/T	17	25.8 (21–33)	fMRI
E2	S/T	17	25.4 (20–35)	fMRI
Lieberman et al., 2017	T	18	22.6 (18–34)	fMRI
Lux et al., 2015	S/T	13	24.8 (21–30)	fMRI
Moscovitch et al., 1995	S	13		PET
Nyberg et al., 1996b	S/T	12	(19–40)	PET
Owen et al., 1996	S	12	26.8 (18–35)	PET
Petersson et al., 2001	S	16	25.0	PET
Rajah et al., 2011	S	16	23.9 (18–34)	fMRI
Rekkas et al., 2005	S/T	10	22.0 (20–37)	fMRI
Ross & Slotnick, 2008	S	12	21.0 (18–35)	fMRI
Slotnick et al., 2003	S	8	(25–45)	fMRI
Suzuki et al., 2005	S	18	21.1 (20–23)	fMRI
Suzuki et al., 2002	T	15	20.7 (19–25)	fMRI
Wang & Diana, 2017	T	17	21.0 (19–32)	fMRI
Zorrilla et al., 1996	T	7	(19–29)	fMRI

fMRI, functional magnetic resonance imaging; PET, positron emission tomography; E, experiment; S, spatial meta-analysis; T, temporal meta-analysis. Empty cells reflect unavailable information.

**Table 2:** Tasks employed in the studies included in the spatial meta-analysis

Bergström et al., 2013	Indicate whether famous faces were presented on the left or right side of the screen.
Burgess et al., 2001	After navigating a virtual town, select the object received in a particular place.
Cansino et al., 2002	Indicate on which quadrant of the screen images of common objects were displayed.
Cansino et al., 2015	Indicate on which quadrant of the screen images of common objects were displayed.
Frings et al., 2006	Indicate whether a cube was located in the same or a different position in a virtual 3D environment that was presented from different perspectives than during encoding.
Hoscheidt et al., 2010	After an autobiographical interview, indicate whether the location or spatial relationship within a personal experience was true or false.
Johnsrude et al., 1999	After pairs of common object drawings were presented with two landmarks or two objects as cues, select the correct location of the object or the correct spatial relationship between objects and cues.
King et al., 2005	After navigating a virtual town, select the object received in a particular place.
Moscovitch et al., 1995	Indicate which of two displays present positions of three common object drawings that were altered from those observed at encoding.
Owen et al., 1996	Indicate which of two positions on the screen was the original location where common object drawings were presented.
Petersson et al., 2001	Indicate which of two positions on the screen was the original location where common object drawings were presented.
Rajah et al., 2011	Select which of three faces was presented in the position of the screen indicated in each trial: left, center, or right. For the difficult condition, order faces from left to right or right to left.
Ross & Slotnick, 2008	Indicate whether abstract shapes were presented on the left or right side of the screen.
Slotnick et al., 2003	Indicate whether abstract shapes were presented on the left or right side of the screen.
Suzuki et al., 2005	Indicate which of two rooms had a photograph presented or whether the photograph was displayed on the left or right side of the screen.

**Table 3:** Tasks employed in the studies included in the temporal meta-analysis

Cabeza et al., 1997	From a pair of words, select the word most recently presented in the study list.
Dobbins et al., 2003	From a pair of words, select the word most recently presented in the study list.
Fujii et al., 2002	Recall the words memorized the day before and those memorized on that morning.
Greve et al., 2010	Select from five options the number of intervening words (5, 10, 15, 20, or 25) between the first and second presentations of the same word in a list.
Lieberman et al., 2017	After videos of store fronts were shown, a reference store was presented with a store-store pair. Select “yes” if they have the same temporal distances from the reference store, and indicate which store was closest in time to the reference store.
Suzuki et al., 2002	Common object drawings were presented in the morning and in the afternoon, three hours after during scanning. From a pair of images, indicate which image was presented in the afternoon, and indicate which image was most recently presented in the list.
Wang & Diana, 2017	Three-word sentences were presented in a familiar order or a randomly scrambled order. Indicate the order of the words as presented during the study.
Zorrilla et al., 1996	From a pair of words, select the word most recently presented in the study list.

**Table 4:** Tasks employed in studies included in both the spatial and temporal meta-analyses

de Rover et al., 2008	Locate the original position of black line drawings in any order within a 3 × 3 grid. Correct positions and correct successive responses in the contiguous grid positions were recorded.
Ekstrom & Bookheimer, 2007	After driving a virtual taxi, select correct passenger-store (destination) pairs and for passenger-passenger pairs, indicate which passenger was delivered first.
Ekstrom et al. 2011	After driving a virtual taxi, a store image was presented followed by store-store pairs. Select which of the two stores was closest in space and time to the first image.
Fujii et al., 2004	After 83 min (on average) of events, select the room where an event occurred and indicate if the event took place before or after a break.
Hayes et al., 2004	After viewing a videotaped tour of the insides of four houses, identify which of two scenes was viewed in the tour and which scene was viewed first.
Kwok et al., 2012	A total of 24 h after watching a 42-min TV episode, select the scene with the same spatial arrangement from a pair of scenes and select the first scene viewed from a pair of scenes.
Kwok & Macaluso, 2015 E1	After watching an average of 10 s of commercial clips, select between pairs of scenes presented and identify the scene with the same spatial arrangement as that in the clip and the first scene viewed.
E2	After watching an average of 10 s of commercial clips, indicate whether the scene had the same spatial arrangement as in the clip and if the scene was presented in the first half or second half of the clip.
Lux et al., 2015	Three months after an autobiographical interview, select from two options to indicate where an event took place and at which age an event occurred.
Nyberg et al., 1996b	Indicate whether words were presented on the left or right side of the screen, and whether words were presented in the first or second list.
Rekkas et al., 2005	Given two autobiographical events, identify which one occurred first and which one occurred further from home.

E, experiment

The specific contrasts employed in each meta-analysis and the number of foci included from each experiment in the spatial and temporal meta-analyses are displayed in Tables 5 and 6, respectively. The total numbers of foci were 487 and 214 for the spatial and temporal meta-analyses, respectively. The contrasts employed to analyze spatial or temporal activity within the brain varied among studies: recognition alone or together with the other context (spatial or temporal) was the contrast most frequently used, followed by comparison with control or baseline measurements or (less often) by comparison with incorrect responses in the same context or another memory task.

**Table 5:** Data included in the spatial meta-analysis

	Contrast(s)	Foci
Bergström et al., 2013	Spatial > Encoding task recollection	6
Burgess et al., 2001	Spatial – Control, Spatial – Person, Spatial – Object	56
Cansino et al., 2002	Spatial correct > Spatial incorrect	17
Cansino et al., 2015	Spatial correct > Spatial incorrect	4
de Rover et al., 2008	Spatial > Baseline; Spatial > Temporal	20
Ekstrom & Bookheimer, 2007	Spatial > Recognition + Temporal	8
Ekstrom et al. 2011	Spatial correct > Spatial incorrect, Spatial > Temporal	15
Frings et al., 2006	Spatial > Control 1, Spatial > Control 2	19
Fujii et al., 2004	Spatial > Recognition, Spatial > Temporal	25
Hayes et al., 2004	Spatial > Recognition	9
Hoscheidt et al., 2010	Spatial > Control	36
Johnsrude et al., 1999	Spatial > Control (four contrasts)^a^	59
King et al., 2005	Spatial > Control, Spatial > Recognition	31
Kwok et al., 2012	Spatial > Recognition + Temporal	16
Kwok & Macaluso, 2015 E1	Spatial > Recognition + Temporal	14
E2	Spatial > Recognition + Temporal, Spatial > Recognition	17
Lux et al., 2015	Spatial > Baseline, Spatial > Temporal	9
Moscovitch et al., 1995	Spatial > Control	11
Nyberg et al., 1996b	Spatial > Recognition	1
Owen et al., 1996	Spatial > Recognition	10
Petersson et al., 2001	Spatial > Baseline	25
Rajah et al., 2011	Spatial > Temporal	13
Rekkas et al., 2005	Spatial – Baseline	4
Ross & Slotnick, 2008	Spatial correct > Spatial incorrect	37
Slotnick et al., 2003	Spatial > Recognition	6
Suzuki et al., 2005	Spatial > Recognition (two contrasts)^a^	19

^a^ Contrasts employed in different spatial memory tasks. E, Experiment.

**Table 6:** Data included in the temporal meta-analysis

	Contrast(s)	Foci
Cabeza et al., 1997	Temporal > Recognition	6
de Rover et al., 2008	Temporal > Baseline, Temporal > Spatial	18
Dobbins et al., 2003	Temporal correct > Temporal incorrect, Temporal > Source	21
Ekstrom & Bookheimer, 2007	Temporal > Recognition + Spatial	15
Ekstrom et al. 2011	Temporal correct > Temporal incorrect, Temporal > Spatial	4
Fujii et al., 2002	Temporal – Control	4
Fujii et al., 2004	Temporal > Recognition, Temporal > Spatial	7
Greve et al., 2010	Temporal correct > Temporal incorrect	15
Hayes et al., 2004	Temporal > Recognition	4
Kwok et al., 2012	Temporal > Recognition + Spatial	3
Kwok & Macaluso, 2015 E1	Temporal > Recognition + Spatial	5
E2	Temporal > Recognition + Spatial	5
Lieberman et al., 2017	Temporal > Baseline (two contrasts)^a^	26
Lux et al., 2015	Temporal > Baseline, Temporal > Spatial	14
Nyberg et al., 1996b	Temporal > Recognition, Temporal > Spatial	2
Rekkas et al., 2005	Temporal – Baseline	4
Suzuki et al., 2002	Temporal > Recognition (two contrasts)^a^	31
Wang & Diana, 2017	Temporal correct > Temporal incorrect	26
Zorrilla et al., 1996	Temporal > Control	4

^a^Contrasts employed in different temporal memory tasks. E, Experiment

**Fig. 2:**
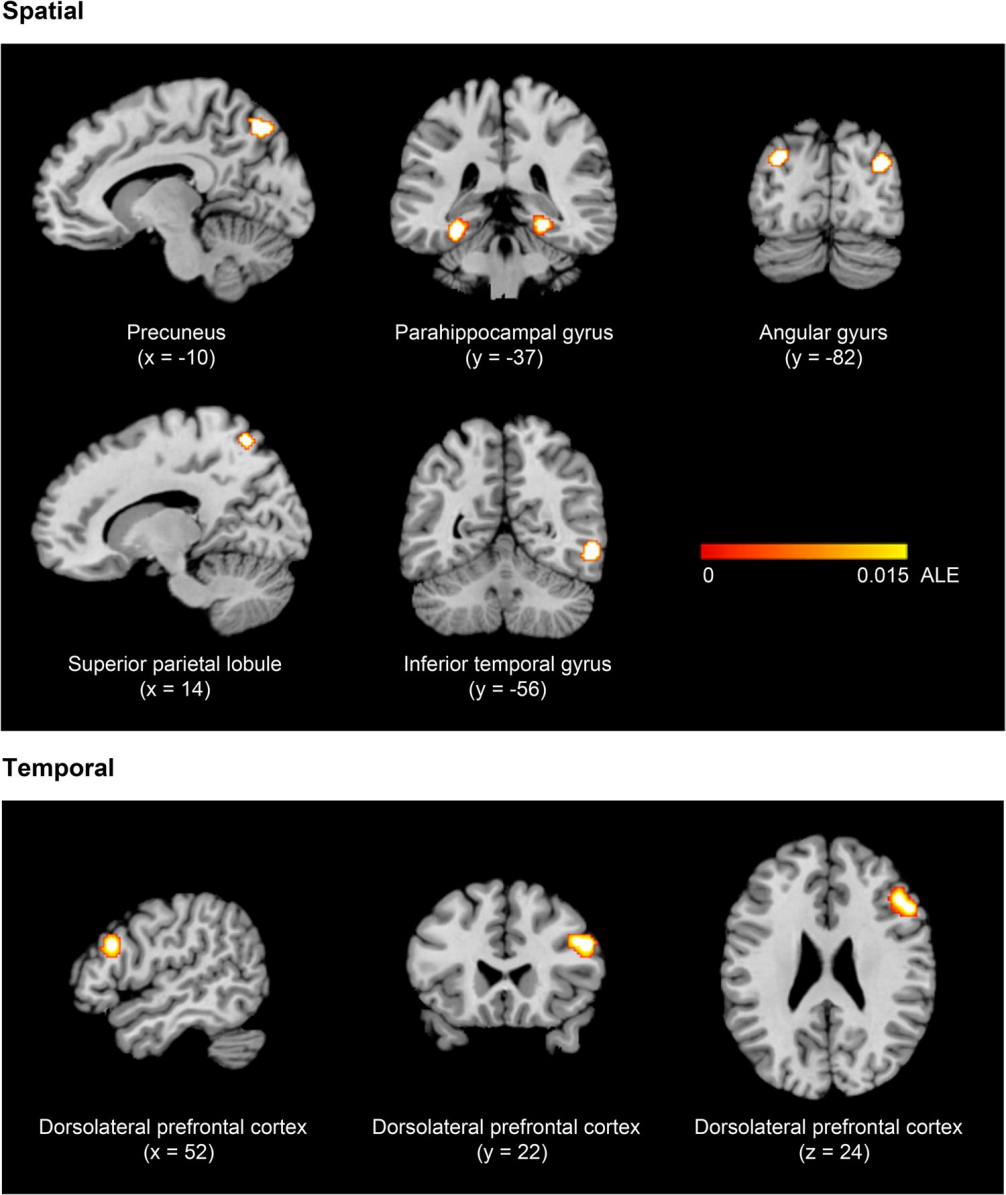
Brain regions exhibiting significant activation in the spatial and temporal meta-analyses. Color bar represents the activation likelihood estimation (ALE) scores

The results of the spatial and temporal ALE meta-analyses are shown in Table 7. The clusters that reached significance in each meta-analysis are displayed in Fig. 2. Seven significant clusters were identified for spatial context retrieval. Two clusters observed in the parahippocampal gyrus and two in the angular gyrus showed symmetrical bilateral activation. The other three clusters were located in the left precuneus, right superior parietal lobule, and right inferior temporal gyrus. Only one cluster revealed significant concurrent activation across studies during temporal context retrieval, located in the right dorsolateral prefrontal cortex.

**Table 7:** Results of the spatial and temporal ALE meta-analyses

Brain region	Hemisphere	BA	MNI coordinates			Volume (mm^3^)	ALE value
			x	y	z		
Spatial							
Precuneus	L	7	−10	−74	48	1400	.0211
	L	7	−18	−68	58		.0174
Parahippocampal gyrus	L	36	−30	−38	-16	1344	.0240
	L	36	−26	−42	-14		.0228
Angular gyrus	R	39	34	−82	26	1120	.0228
Superior parietal lobule	R	7	14	−64	60	1024	.0200
	R	7	2	−66	58		.0159
Inferior temporal gyrus	R	37	56	−56	−8	1000	.0236
Angular gyrus	L	39	−30	−82	30	1000	.0244
	L	39	−32	−72	38		.0163
Parahippocampal gyrus	R	36	22	−36	−12	912	.0197
	R	36	26	−38	−12		.0194
	R	36	30	−46	−14		.0167
Temporal							
Dorsolateral prefrontal cortex	R	9	52	22	24	2152	.0200
	R	9	46	28	24		.0183

L, left; R, right; BA, Brodmann’s area; ALE, activation likelihood estimation

## Discussion

The main findings were as follows. Two regions involved in the retrieval of spatial information exhibited marked activation symmetry in the two hemispheres: the parahippocampal gyrus and the angular gyrus. Likewise, regions in the left precuneus, right superior parietal lobule, and right inferior temporal gyrus contributed to the retrieval of spatial information. Only one cluster in the right dorsolateral prefrontal cortex was responsible for the retrieval of temporal information. Below, we discuss these findings in detail.

Contrary to our predictions, the spatial and temporal meta-analyses failed to identify any significant hippocampal regions. This is possibly because only nine of the 26 experiments included in the spatial meta-analyses (Burgess et al., 2001; Cansino et al., 2002, 2015; Ekstrom et al., 2011; Hoscheidt et al., 2010; Johnsrude et al., 1999; King et al., 2005; Lux et al. 2015; Ross & Slotnick, 2008) reported hippocampal activation in whole-brain analyses related to the retrieval of spatial contextual information. For the temporal meta-analysis, only four of 19 experiments (Ekstrom et al., 2011; Lieberman et al., 2017; Lux et al. 2015; Wang & Diana, 2017) reported hippocampal activation during temporal context retrieval. A previous meta-analysis conducted to identify associations between brain activity and objective recollection also failed to find hippocampal activation (Spaniol et al., 2009). Two additional reasons could explain the difficulty in detecting hippocampal activity associated with episodic retrieval (Stark & Squire, 2000). One reason is the hippocampus’s small size and proximity to the sinus cavity. The other reason is that when comparing targets with foils, the activation differences between the two are diluted because unfamiliar foils tend to be encoded, eliciting hippocampal activation. Although hippocampal activity was not observed in the current study, we assume that it contributes to the processing of spatial and temporal information given substantial evidence of its support, as reviewed in the introduction. Regarding episodic memory, it is well established that the hippocampus is critical for recollection (for review, see Eichenbaum, 2017a). Recollection refers to the ability to retrieve events accompanied by contextual information, whereas familiarity is defined as the ability to remember the event without accompanying contextual information (Mandler, 1980); thus, only the former is truly episodic. Moreover, the hippocampus coordinates brain regions involved in recollection encoding and retrieval, as revealed by its connectivity with almost all regions engaged in these processes (for review, see Cansino, 2022).

Notably, different regions supported the independent retrieval of spatial and temporal information. This finding indicates that beyond the hippocampus, where certain regions process both spatial and temporal information, as revealed by studies in rats (Kraus et al., 2013) and humans (Deuker et al., 2016; Nielson et al., 2015), the retrieval of spatial and temporal information followed different routes. Spatial information processing is attributed to the dorsal stream (the “where” pathway) that projects from the primary visual cortex to the posterior parietal cortex, as opposed to the ventral stream (the “what” pathway) that processes visual object information, which projects from the primary visual cortex to the inferior temporal lobe (Mishkin & Ungerleider, 1982). However, temporal information processing is not associated with any neural pathway, in contrast to spatial and visual object information. This may be attributed to the fact that spatial and temporal information are not equivalent attributes of episodic memory.

To illustrate this argument, imagine traveling overseas to an unfamiliar city. After a certain amount of time, when you attempt to retrieve the details of the trip, you likely would be able to reconstruct the spatial characteristics of the places visited, but you may not be able to retrieve any physical representation of the time related to your trip. Therefore, space is linked to a physical representation, whereas time is linked to an abstract representation that is built up by sequences of information. However, space also may be conceived as merely a mental inference because what is preserved in memory is a relative representation of the actual physical experience. Friedman (1993) proposed that there are three types of temporal memory: remembering the time elapsed between the event and the present, the identification of the event within a particular period, and the order of the event in relation to other events. Thus, temporal information is the product of mental inference and is not related to any concrete representation. Another crucial difference between space and time is that time proceeds only in one direction, from the past into the future, as proposed by Eddington (1928) in his time’s arrow concept. The asymmetrical direction of time has no equivalence in the spatial dimension. Spatial environments can be revisited, but we cannot go back to the past, except through mental traveling. These dissimilarities between spatial and temporal information may explain why the brain regions responsible for the retrieval of each of these types of contextual information differ.

### Spatial recollection

Spatial context recollection is supported by a brain cortex network comprised of the parahippocampal gyrus, angular gyrus, precuneus, superior parietal lobule, and inferior temporal gyrus. Notably, the parahippocampal gyrus and angular gyrus showed marked symmetry of activity in the two hemispheres. The parahippocampal gyrus is situated in the medial temporal lobe, surrounding the hippocampus, and has afferent and efferent connections with the hippocampus and all the other regions that contribute to spatial recollection (Van Hoesen, 1982). The angular gyrus situated in the posterior part of the inferior parietal lobule connects to the hippocampus (Uddin et al., 2010), parahippocampal gyrus (Rushworth et al., 2006), precuneus (Makris et al., 2007), superior parietal lobule, and inferior temporal gyrus (Petit et al., 2023). The precuneus is located in the medial posterior parietal cortex and has bidirectional connections with all the other regions responsible for spatial information retrieval (Tanglay et al., 2022; Lin et al., 2020), in addition to the hippocampus (Wang et al., 2019). The superior parietal lobule, located in the posterior parietal cortex, is a highly connected region; it is connected to all the other regions involved in spatial recollection and the hippocampus (Lin et al., 2021; Rockland & Van Hoesen, 1999). Moreover, the inferior temporal gyrus is connected to all regions in the spatial retrieval network, in addition to the hippocampus (Lin et al., 2020). The physical connections among regions in the spatial retrieval network indicate that it is plausible that these regions work together. Indeed, all the regions that encompass this network are connected to each other and have a direct connection with the hippocampus.

Three of the spatial network regions identified in the present study—the parahippocampal cortex, the angular gyrus, and the superior parietal lobule (the latter two located in the posterior parietal cortex)—are considered part of the core recollection network, along with the hippocampus, anterior medial prefrontal cortex, and posterior cingulate (Yonelinas et al., 2005); this network is considered to underlie recollection, independent of stimulus type or retrieval task (Rugg & Vilverg, 2013). The crucial role of the parahippocampal cortex and posterior parietal cortex in recollection has led to the formulation of highly influential models of how they contribute to recollection. Below, we review some of these models and discuss how they relate to contextual recollection. The precuneus has been linked with spatial processing, but not exclusively, and the inferior temporal gyrus has been linked with object identification. Below, we discuss the evidence that supports the functional properties of these regions. The spatial network observed in the current study is closely related to previous findings and provides a clear view of how spatial information retrieval might be accomplished, as we outline at the end of this section.

One of the first fMRI studies (Epstein & Kanwisher, 1998) that detected parahippocampal activation during spatial processing observed that a specific area within this region was activated when participants passively observed scenes but not when they viewed single objects or faces. Thus, this area was named the “parahippocampal place area,” because its activity was related to the ability to process spatial environmental information. Subsequent research has revealed that the parahippocampal gyrus also processes spatial information to build episodic memory representations within specific spatial contexts, as well as all types of contextual associations within episodic memory (for review, see Aminoff et al., 2013).

Several models have been proposed to explain how the parahippocampus contributes to recollection. For example, an influential framework (Eichenbaum et al., 2007) proposes that contextual information, especially spatial information, is represented in the parahippocampal cortex and transmitted to the hippocampus to be integrated with the event into an episodic representation. Then, during retrieval, the hippocampus mediates the recollection of the contextual representation allocated in the parahippocampal cortex. Likewise, the “binding of item and context” model (Diana et al., 2007) proposed that the posterior parahippocampal cortex processes contextual information, not only spatial information, and item-context associations occur in the hippocampus. During retrieval, the hippocampus reactivates the contextual information trace in the parahippocampal cortex to achieve recollection. Another model (Ranganath & Ritchey, 2012) proposes the existence of the anterior temporal system and posterior medial system. The core component of the former system is the perirhinal cortex, which supports familiarity and assessing the importance of entities. The core components of the posterior medial system are the parahippocampal cortex and retrosplenial cortex. This system supports recollection through the construction of mental representations that integrate the entities and multiple contextual environmental features present during an experience.

All of these models share the view of the parahippocampal cortex as the key region involved in processing contextual information to support the integrative role of the hippocampus in recollection. Therefore, the identification of the parahippocampal cortex as part of the spatial retrieval network in the current study is highly expected. Moreover, the cluster we observed in the parahippocampal gyrus is situated in a posterior portion of this region, a location that is in agreement with previous studies and models (Diana et al., 2007; Epstein & Kanwisher, 1998; Ranganath & Ritchey, 2012). Moreover, bilateral activation of the parahippocampal cortex has been previously observed regarding spatial context (Ekstrom & Bookheimer, 2007) and object context (Li et al., 2016), indicating that bilateral activation is a characteristic of associative memory. However, this also shows that episodic memory does not rely on a single hemisphere; as it is a highly complex process, it requires the involvement of both hemispheres to ensure memory reconstruction. The bilateral activation observed in the angular gyrus also reflects this reinforcement mechanism for guaranteeing recollection.

The posterior parietal cortex, which includes the angular gyrus and superior parietal lobule, has been strongly linked to spatial processing. Space is considered a multimodal experience, because it can be estimated by multiple sensations. Spatial locations may be recognized by sight, sound, and touch (Colby & Goldberg, 1999). Additionally, the experience of space is not represented by a single map within the brain but by multiple representations that dynamically vary as a consequence of the individual’s movements. Moreover, multiple spatial representations of the environment are encoded in the posterior parietal cortex (Colby & Goldberg, 1999). Further research has revealed that the posterior parietal cortex also has a critical role in spatial attention and episodic memory (Wagner et al., 2005). Several models have been proposed to explain how the posterior parietal cortex contributes to episodic memory. Notably, most of the models propose that within the posterior parietal cortex, there are two functionally distinguishable regions: the dorsal posterior parietal cortex, which includes the superior parietal lobule and intraparietal sulcus, and the ventral posterior parietal cortex, which includes the marginal gyrus and angular gyrus, also known as the inferior parietal lobule (Cabeza et al., 2008; Shimamura, 2011; Vilberg & Rugg, 2008).

For example, in the “attention to memory” model (Cabeza et al., 2008), the posterior parietal cortex contributes to episodic memory retrieval through attention processes. In particular, top-down attention and bottom-up attention are allocated by the dorsal and ventral portions of the posterior parietal cortex, respectively. According to this model, top-down attention is triggered when the information needed to retrieve the episodic memory event is insufficient and further guided memory search is needed. Familiarity is mainly associated with top-down attention, because further information is needed to reach a memory decision. Conversely, bottom-up attention is associated with recollection because the spontaneous recovery of relevant cues or contextual details activates bottom-up attention. The episodic buffer theory (Vilberg & Rugg, 2008) suggests that the dorsal posterior parietal cortex is not involved in episodic memory but in processing stimulus salience or stimuli that are task relevant. In contrast, the ventral posterior parietal cortex is involved in recollection through operations similar to those attributed to the “episodic buffer” proposed by Baddeley (2000). Thus, this region contributes to recollection by recovering, maintaining, and integrating episodic representations online with the assistance of executive functions. The “cortical binding of relational activity” model (Shimamura, 2011) views the dorsal posterior parietal cortex as a component of the dorsal path specialized for selective attention and the processing and storage of events’ spatial attributes. The ventral posterior parietal cortex binds multimodal features distributed across the neocortex related to an episodic event, which supports reinstatement and, therefore, recollection.

The spatial brain network identified in the current study included clusters in the superior parietal lobule and angular gyrus, regions that correspond to the dorsal and ventral posterior parietal cortex, respectively. The models outlined above mostly align with the present findings, with a few exceptions. One of these exceptions is that the activity in the superior parietal lobule occurred during successful spatial recollection; therefore, the top-down attention arising from this region may not be associated with familiarity. Indeed, the present findings revealed that recollection requires both top-down attention and bottom-up attention depending on the amount of information available during retrieval. The allocation of top-down attention led to spatial recollection and not merely to familiarity. The present findings do not support the proposal that the superior parietal lobule responds to stimulus salience or stimuli that are task relevant because, although the diverse recollection tasks employed by the studies included in the meta-analysis did not manipulate these attributes, the superior parietal lobule was relevant for spatial recollection. The present results may be interpreted according to any of these models, because all of them apply to the current outcome, and it is highly probable that all the functions attributed to the posterior parietal cortex actually occur depending on the circumstances in which recollection is achieved.

The precuneus is an association area involved in several cognitive functions, such as generating allocentric and egocentric spatial relations, guiding attention to spatial locations, shifting attention among spatial locations and performing episodic memory retrieval (for review, see Cavanna & Trimble, 2006). One of the first pieces of evidence demonstrating the contribution of the precuneus to episodic memory recall was reported in a PET study (Fletcher et al., 1995) that compared cue recall for imaginary and nonimaginary word pairs. The former elicited bilateral precuneus activation that led the authors to label this region the “mind’s eye,” because its activity was clearly associated with visual imagery processes during episodic memory retrieval. Further research confirmed that the role of the precuneus in episodic memory extends beyond mental imagery. For example, activation of the precuneus has been observed during the reinstatement of contextual associations (Lundstrom et al., 2003), the retrieval of context-rich memories (Gilboa et al., 2004), and the retrieval of specific personal experiences (Addis et al., 2004). Therefore, the results of the current study further confirmed the crucial role of the precuneus in spatial contextual recollection, a type of context that requires the contribution of a region specialized in the ability to guide attention to locations and mentally visualize those locations to enable their successful recollection.

The posterior parietal cortex and the inferior temporal cortex are the final locations of the dorsal and ventral streams, respectively (Mishkin & Ungerleider, 1982). The attributes of a visual object, such as its size, color, texture, and shape, are integrated in the inferior temporal cortex to form an object representation, which, once identified by memory processes, is stored as a general representation (Miyashita, 1993), which is a representation that allows the identification of an object independent of the viewer’s perspective (Perrett et al., 1985). Thus, the inferior temporal cortex contributes to visual perception and object recognition. The functional attributes of this region were confirmed by the fact that bilateral excision of the inferior temporal cortex severely impairs visual object recognition (Mishkin, 1982). Therefore, the inferior temporal cortex within the spatial brain network may play a role in recognizing the event or object with which the spatial context is associated. The spatial context is an attribute of events; thus, spatial information cannot be processed independently of the event.

All regions involved in spatial recollection were association areas within the cerebral cortex, which do not receive direct sensory input or provide motor output but are responsible for the complex integrations of visual, auditory, and somatosensory information (Pandya & Seltzer, 1982). In addition to having integrative functions, all of these regions (except the inferior temporal gyrus) process different spatial features. The inferior temporal gyrus recognizes objects or events; then, to retrieve the spatial context associated with the event, the brain performs several functions, such as the reconstruction of the episodic experience by integrating the event within its spatial context (parahippocampal cortex); allocation of top-down attention and bottom-up attention toward relevant recovery cues and memories to guide spatial recollection (posterior parietal cortex); the recovery, integration and maintenance of episodic representation online with the assistance of executive functions (posterior parietal cortex); the binding of spatial information distributed across the neocortex into the episodic representation endorsing reinstatement (posterior parietal cortex); the internal direction of attention to different spatial locations, and the mental imagery of the spatial environment related to the episode (precuneus).

### Temporal recollection

The dorsolateral prefrontal cortex was the only region that contributed to the retrieval of temporal contextual information. This region situated in the middle frontal gyrus is connected to the hippocampus (Barbas & Blatt, 1995). To estimate when an event took place, the brain has to perform a series of high-level cognitive processes, such as elaborating on the experience to deduce its date, determining associations with other information or events that might serve as clues, organizing a sequence of related events and reconstructing their relative order of occurrence. Clearly, all these processes depend on executive functions attributed to the dorsolateral prefrontal cortex (Elliot, 2003), which explains the involvement of this region in the retrieval of temporal information. Executive functions encompass many other functions, such as working memory, task switching, planning, organization, decision-making, and inhibitory control (Elliot, 2003). These functions are essential for any higher-order cognitive process, including episodic memory. For example, through executive functions, such as working memory, the dorsolateral prefrontal cortex may contribute to the organization of information from an episodic event to estimate and recollect its timing.

Moreover, the dorsolateral prefrontal cortex also participates in episodic memory. Early studies consistently observed that during encoding, the left dorsolateral prefrontal cortex exhibited greater activation, whereas during retrieval, the right dorsolateral prefrontal cortex exhibited greater activation (Fletcher et al., 1998). During encoding, this activity was interpreted as the consolidation of an episodic structure that emphasizes the abstract meaning of the episode. Then, at retrieval, the episodic structure is used to mentally control and monitor the recovered episode (for review, see Nyberg et al., 1996a). Later studies reported that the dorsolateral prefrontal cortex also contributes to retrieving specific details associated with the events (Wagner et al., 1998), performing internal decisions that occur before providing a response (Hayama & Rugg, 2009), and monitoring the postretrieval results of a retrieval attempt (Rugg et al., 2002). A review of several fMRI studies concluded that the dorsolateral prefrontal cortex was activated during the selection, manipulation, and monitoring of episodic memory (Fletcher & Henson, 2001). Thus, the dorsolateral prefrontal cortex might contribute to episodic memory through any of the functions outlined above, and their selection seems to depend on the retrieval task and experimental conditions.

All functions attributed to the dorsolateral prefrontal cortex could support the retrieval of temporal context information, but not exclusively temporal information; these functions may be relevant for the recollection of any type of contextual information. However, in the present study, we found that this region is crucially involved in the retrieval of the temporal context associated with an episodic event, because it was the most robustly implicated region across studies. The activity observed in the dorsolateral prefrontal cortex was associated with the accurate retrieval of temporal information, indicating that this region participates in more than a monitoring process independent of retrieval success. The executive functions that are also attributed to the dorsolateral prefrontal cortex, particularly working memory, seem to be essential for the mental reconstruction of temporal information associated with an episodic experience. Likewise, the activity observed in the dorsolateral prefrontal cortex may reflect the increase in top-down control of retrieval processes, because the abstract nature of temporal information involves more difficult and demanding retrieval operations. Top-down control of retrieval processes has been described as a mechanism guided by retrieval goals, supported by the interaction between the prefrontal cortex and hippocampus (Dobbins et al., 2002). Therefore, the present findings revealed that among the brain regions (other than those in the medial temporal lobe) involved in the retrieval of temporal context information, the dorsolateral prefrontal cortex is the most relevant.

Although the dorsolateral prefrontal cortex was the only region identified in the temporal meta-analyses, as mentioned for the hippocampus, we assume that other regions are also relevant for temporal context retrieval. In particular, the parahippocampal cortex is important, because according to several models (Diana et al., 2007; Ranganath & Ritchey, 2012) this region processes all types of contextual information and helps the hippocampus to integrate complex episodic memory representations to achieve recollection. The reason that this relevant region was not noted in the temporal meta-analysis may be because only three of 19 experiments (Lux et al., 2015, Rekkas et al., 2005; Wang & Diana, 2017) reported activity in this region; therefore, the original studies themselves failed to detect parahippocampal activation, preventing it from being detected as a relevant brain region in the current study.

## General remarks

A brain network that encompasses seven brain regions was identified as essential for recollecting the spatial context of episodic events, whereas a single bran region was relevant for recollecting the temporal context associated with an event. The finding that only one region was critical for temporal recollection could be explained by the fact that the temporal context has to be reconstructed through several processes and inferences. For example, such information could be reconstructed by retrieving and analyzing concomitant events or events that occurred before or after the event of interest to estimate the timing of the relevant episode. Therefore, the surrounding events that are employed as clues could be extremely varied, leading to the recruitment of diverse brain regions depending on the nature of the clues. The variable brain regions supporting recollection of a temporal context preclude the identification of systematic patterns of activation across studies to detect the other brain regions involved in temporal recollection.

One important characteristic of the studies included in the temporal meta-analysis is that approximately 53% employed words as stimuli. However, only 19% of the studies included in the spatial meta-analysis used words as stimuli. The fact that most of the studies in the latest meta-analysis used images led to greater homogeneity among studies; therefore, more regions could be detected regarding the retrieval of spatial context. The power to detect regions involved in the retrieval of the temporal context was diluted by the employment of almost the same proportions of studies using words and images. Moreover, there is clear evidence that images are more memorable than words, because images generate a dual code (verbal and image codes), whereas words only generate a verbal code (Paivio et al., 1975) or require further effort to generate another code. Thus, different processes underlie the retrieval of each type of stimulus. The low number of experiments that examined temporal context retrieval prevented us from performing additional meta-analyses to examine whether the type of stimulus had a direct influence on our results.

Another factor contributing to the variability in (and therefore the difficulty of detecting) concurrent activation across studies during temporal context retrieval could be the different times elapsed between the encoding and retrieval phases. There is evidence (Furman et al., 2012) that memory accuracy and brain activity decline with time. Most studies examined retrieval immediately after encoding, but others examined retrieval after more than 1 hr (Fujii et al., 2004), after several hours (Fujii et al., 2002; Suzuki et al., 2002), after 1 day (Fujii et al., 2002; Kwok et al., 2012), or after years, such as memory of autobiographical events (Lux et al., 2015; Rekkas et al., 2005). The time between encoding and retrieval for examining spatial context also varied among studies. Five studies used long lapsed times (Fujii et al., 2004; Hoscheidt et al., 2010; Kwok & Macaluso, 2015; Lux et al., 2015; Rekkas et al., 2005). However, the elapsed time variability did not prevent the identification of several activation clusters supporting spatial context retrieval, as for temporal recollection analyses. This variability may have stronger effects if fewer studies are included in the meta-analysis, as it arose in the temporal meta-analysis but not the spatial meta-analysis.

A limitation of the present study is that few studies were included in the temporal meta-analysis (19 experiments from 18 studies fulfilled our inclusion criteria) compared with the spatial meta-analysis (26 experiments from 25 studies). Although the number of experiments included in both meta-analyses was more than 17, which corresponds to a statistical power of 80% (Eickhoff et al., 2016), the two meta-analyses did not have equivalent statistical power due to the different number of studies included. A satisfactory statistical power is needed to detect smaller effects and to ensure that the results are not influenced by only one or two of the experiments (Müller et al., 2018). This limitation highlights the importance of further research on temporal context recollection to confirm the current findings. Nevertheless, we believe that we were able to provide essential information about the underlying brain regions responsible for spatial and temporal recollection. Another limitation is that the results of the studies included in the meta-analyses are in essence correlative and not casual. Therefore, the meta-analysis results are also correlative, and additional research using other statistical approaches and complementary techniques is needed to determine causal relationships.

## Conclusions

Spatial retrieval involves the following brain regions: the inferior temporal gyrus, which identifies the episodic event; the parahippocampal cortex, which integrates the spatial context with the episodic event; the superior parietal lobule, which employs top-down attention to search internally for relevant cues; the angular gyrus, which engages bottom-up attention guided by spontaneous recovery cues, performs executive functions to recover, integrate and mentally maintain the episodic representation, and conducts binding to integrate the spatial context dispersed across the neocortex; and the precuneus, which mentally reestablishes the spatial contextual environment. Temporal retrieval requires the participation of the dorsolateral prefrontal cortex, which supports the complex executive functions needed to estimate the temporal context associated with the event. Together, these regions enable the best recollection of an episodic memory that includes the most relevant attributes of any personal experience in terms of the location and time of occurrence.

## Data Availability

Not applicable.
